# Effect of Intra- and Interspecific Competition on the Performance of Native and Invasive Species of *Impatiens* under Varying Levels of Shade and Moisture

**DOI:** 10.1371/journal.pone.0062842

**Published:** 2013-05-10

**Authors:** Hana Skálová, Vojtěch Jarošík, Šárka Dvořáčková, Petr Pyšek

**Affiliations:** 1 Institute of Botany, Academy of Sciences of the Czech Republic, Průhonice, Czech Republic; 2 Department of Ecology, Charles University in Prague, Prague, Czech Republic; USDA-ARS, United States of America

## Abstract

Many alien plants are thought to be invasive because of unique traits and greater phenotypic plasticity relative to resident species. However, many studies of invasive species are unable to quantify the importance of particular traits and phenotypic plasticity in conferring invasive behavior because traits used in comparative studies are often measured in a single environment and by using plants from a single population. To obtain a deeper insight into the role of environmental factors, local differences and competition in plant invasions, we compared species of *Impatiens* (Balsaminaceae) of different origin and invasion status that occur in central Europe: native *I. noli-tangere* and three alien species (highly invasive *I. glandulifera*, less invasive *I. parviflora* and potentially invasive *I. capensis*). In two experiments we harvested late-stage reproductive plants to estimate performance. The first experiment quantified how populations differed in performance under varying light and moisture levels in the absence of competition. The second experiment quantified performance across these environments in the presence of intra- and inter-specific competition. The highly invasive *I. glandulifera* was the strongest competitor, was the tallest and produced the greatest biomass. Small size and high plasticity were characteristic for *I. parviflora*. This species appeared to be the second strongest competitor, especially under low soil moisture. The performance of *I. capensis* was within the range of the other *Impatiens* species studied, but sometimes limited by alien competitors. Our results suggest that invasion success within the genus *Impatiens* depends on the ability to grow large under a range of environmental conditions, including competition. The invasive species also exhibited greater phenotypic plasticity across environmental conditions than the native species. Finally, the decreased performance of the native *I. noli-tangere* in competition with other species studied indicates that this species may be possibly excluded from its sites by invading congeners.

## Introduction

Invasive species represent a major threat to native biodiversity and the functioning of invaded ecosystems [1]–[4] due to suppressing resident native plants [5]–[8]. The factors underlying successful invasions have been intensively studied (e.g. [9]–[12]) and include studies that have characterized the traits that distinguish successful invaders from those that failed, or native species [13], [14]. In these studies, the comparisons of closely related species were shown to be a convenient tool for identifying mechanisms linked with plant invasiveness since by using this approach, phylogenetic biases are minimized [14]–[17].

Tall stature, high growth rate, high fecundity, good survival and efficient dispersal are repeatedly reported as traits associated with invasive plant species, in comparison with native species and non-invasive congeners [13], [18], [19]. In addition, it is suggested that invasive plants are often phenotypically plastic, which enables them to grow and reproduce in a wide range of environmental conditions [20]–[22] and broaden their habitat niche in the invaded range [23], [24]. Greater levels of plasticity have been found in some invasive plants compared to their native or non-invasive congeners [11], [25], [26]. However, this pattern does not hold for all invasive species and traits [27] and its importance depends on the specific environment. The plasticity of morphological or physiological traits can contribute to invasion success if it provides the invader with improved ability to optimize fitness in a variety of environments and/or take advantage of favourable environments [23]. Thus, measuring plant traits in more than one physical environment [28]–[30] or under competition [31] can provide important insights into the role of plasticity in plant invasions. Locally adapted genotypes developing during the initial lag phase following introduction into a new region [32] have also been shown to play a role in the invasion process [33]–[35]. However, the role of phenotypic plasticity and genetic differentiation in plant invasions started to be simultaneously addressed only recently [36]–[38].

Most studies comparing the performance of closely related invasive and native species in response to environmental factors are based on cultivation experiments without competition. However, abundances of species in a community result from interspecific interactions, which can differ along environmental gradients [39], as can the competitive advantage of invasive plants [40], [41]. Despite this, studies comparing the response of closely related invasive and native species to environmental factors in the presence of a competitor have started to appear only recently, and have imposed competition under varying levels of a single environmental factor such as nitrogen [42]–[44] or light [45]. One study compared an invasive species with its common and rare native congeners [44] but research on the above issues and including congeners with different invasion status has been lacking.

We explored the role of phenotypic plasticity and local differentiation in shaping performance outcomes in *Impatiens* (Balsaminaceae) of varying origin and invasion status (following the terminology outlined in [46]–[48]). The species studied were annuals occurring in central Europe: the native *I. noli-tangere* and three aliens differing in their invasion status: the highly invasive *I. glandulifera*, less invasive *I. parviflora* and potentially invasive *I. capensis* [38], [49]. These species coexist in some sites; this makes it possible to minimize habitat- and community-related biases because species from the same habitats tend to be similar to each other in terms of ecology, and different from species in other habitats [50], [51]. In particular, *I. noli-tangere* and *I. parviflora* may come more often into competition with *I. glandulifera* in the near future because the latter spreads from river banks into the surrounding habitats where the former two typically occur [52]; such competition between closely related species is assumed to be particularly strong [53]. In this study, consisting of two experiments, we ask: (i) Are there any differences in traits of adult plants of the species? (ii) How do the traits of plants respond to experimental manipulation of environmental factors? (iii) Do the species differ in plasticity in response to shading, water availability and competition with congeners? (iv) Is there any local differentiation of populations originating from different localities?

## Methods

### The species studied

The *Impatiens* species studied have similar life-histories and reproductive characteristics, and they coexist in some habitats [54]–[57]. *Impatiens noli-tangere* is native to the Czech Republic [58]. The two species invasive in the Czech Republic, *I. glandulifera* and *I. parviflora* [59], were planted in Europe as garden ornamentals and escaped from cultivation at similar times in the mid 19th century [60]. *Impatiens glandulifera* is rapidly spreading in the Czech Republic [38], [49], [52], [61], while the spread of *I. parviflora* is wider but more stabilized [62]. *Impatiens capensis* is invasive in Western Europe and the closest localities to the Czech Republic are central Germany [49]. As annuals with a limited seed bank [63], the population maintenance of all the studied *Impatiens* species depends on successful performance every year.

### Localities and seed collection

Seeds were collected in July and August of 2008 and 2009. In the Czech Republic, localities that harboured all three *Impatiens* species present in the country were chosen. Seeds of *Impatiens glandulifera*, *I. parviflora* and *I. noli-tangere* were collected from the following localities: Černětice near Volyně (coded as VOL; N 49°8′30″, E 13°53′50″), Velký Osek (POL; N 50°07′, E 15°10′) and Paskov (PAS; N 49°44′, E 18°18′). For collections made at VOL, all three species grew in mixed stands or very close to each other in forests, or along shaded brooks and river banks. At POL and PAS, seeds of *I. noli-tangere* and *I. parviflora* were collected from mixed forest stands and those of *I. glandulifera* along a river bank within 1–2 km. Seeds of *I. capensis* were collected from three localities in central Germany (region of Frankfurt am Main) in September 2008: along the banks of a brook in open farmland near the village of Großseelheim (GRV; N 50°48′37″, E 8°52′52″), in a wet pasture between the villages of Großseelheim and Schröck (GRP; N 50°48′6″, E 8°50′15″) and in wet meadow margins and a spruce forest near the homestead village Hassemülle (HAS; N 50°12′8′, E 8°21′20″).

For each species, we collected a mixed sample of seed from at least 100 individuals randomly chosen from the whole site. The seeds were sown into pots filled with common garden soil in October and kept in the experimental garden until seedlings appeared that were used for the experiments. The identity of seeds from the individual localities was used in the first experiment examining single species. For the second experiment examining intra- and interspecific competition we used seeds collected from POL and in *I. capensis* from plants used in the first experiment.

For any locations/activities specific permission was not required as the species and the areas are not protected or privately owned as well as the study did not treat endangered or protected species.

### Experiments

The performance of the species was assessed in two greenhouse experiments lasting from the juvenile to senescent life stages. In the first experiment, we studied plant performance in response to environmental factors and in relationship to local differentiation. In the second experiment, we quantified the response to the environmental factors under intra- and interspecific competition.

Both experiments started in May when randomly chosen seedlings were transplanted into plastic 19×19×23 cm pots filled with common garden soil. The pots were placed in a greenhouse with two shade and moisture levels in a factorial design. Shade levels were achieved by using a green garden shading net transmitting 35% and 90% of incident radiation (hereafter referred to as mild and deep shade, respectively). Moisture levels were maintained by using a micro-drip system (Hunter Industries, San Marcos, USA). The plants were supplied by tap water provided into each pot. Soil moisture was measured in each pot in 7–14 day intervals using an HH2 Moisture Meter device equipped with a ThetaProbe ML2x Soil Moisture Sensor (Delta-T Devices, UK). The micro-drip system was then readjusted to keep soil moisture at 20% and 40% (hereafter referred to as low and high moisture, respectively). Due to logistic reasons, particular species in Experiment 1 (and species pairs in Experiment 2) subjected to the moisture levels were placed in blocks within the shading treatments.

In the first experiment, we planted one plant per pot. A total of 480 plants were grown, consisting of 10 replicates per species (4), locality (3) and treatment (4). Plants in the second experiment were planted in pairs, including pairs of the same species to reveal the effect of intraspecific competition. A total of 800 plants were planted into 400 pots, with 10 pots per species pair type (10) and treatment (4). Some plants did not survive until the end of the experiments, but the number of individuals did not decrease below 8 (i.e. 80% survival) in any of the groups. Thus, the total number of analyzed plants after accounting for mortality was 465 in the first experiment and 768 in the second experiment.

The plants were harvested when symptoms of senescence appeared, i.e. between mid-August and the end of September. Senescence occurred earlier in *I. parviflora* and *I. noli-tangere*, and in low-moisture treatments. In both experiments, stem height, number of first-order lateral branches (hereafter referred to as branches) and shoot biomass were recorded at the time of harvest. In the first experiment, biomass was separated into the main stem (hereafter referred to as stem), branches, leaves and peduncles. The biomass of peduncles was used as a proxy for reproductive effort; this is justified because the seeds are produced during most of the growing season and released immediately after maturation, but peduncles remain attached to the stems. The biomass was dried at 60°C for about 6 hours and weighed.

### Data analysis

The first experiment was evaluated by linear mixed effect models (LMMs). Stem height, branch number, shoot biomass, peduncle biomass, stem biomass, branch biomass and leaf biomass were the response variables. Two levels of shade (deep and mild) and soil moisture (high and low) and the identity of the four species (*Impatiens capensis*, *I. glandulifera*, *I. noli-tangere* and *I. parviflora*) were the fixed factorial treatment effects, and the six localities a random effect, in all the mixed models. In mixed models testing allocation of biomass, the biomass of stem, branch, leaf and peduncle were held constant by adding shoot biomass as a covariate, allowing this covariate to change specifically for identity of each species. To evaluate the models, top-down strategy for model selection process in LMMs [64] was applied, following Zuur et al. ([65], p. 120–129). The modeling started with the beyond optimal model, where the fixed component contained all explanatory variables and their interactions. As a first step, whether the random component locality is necessary was assessed. This was done by likelihood ratio (LR) test on nested models, comparing a model without a random effect with a model containing locality as a random intercept. The nested models were evaluated by restricted maximum likelihood method (REML), obtaining the correct probability values following Verbeke and Molenbergh [66]'s testing on the boundary. Once the optimal random structure has been found, as a second step the optimal fixed structure was examined. The aim was to determine the minimal adequate model (MAM). In MAM, all explanatory variables were significantly different from zero and from one another, and all non-significant explanatory variables were removed using a step-wise process of model simplification (e.g. [67]). This process began with the maximal model containing all the fixed explanatory variables and their interactions, and continued by the elimination of non-significant terms using deletion tests. To prevent biases to the model structures caused by correlation between variables, model simplifications proceeded via a backward elimination from the maximal models using step-wise analysis of deviance tables (e.g. [68], p. 192–197). The results were thus not affected by the order in which the explanatory variables were removed in the step-wise process of model simplification. The final models with the optimal random and fixed structure were presented using REML. From these models, in case of significant random effects of localities, intraclass correlations (ICC, e.g. [65], p. 138) were calculated, to assess associations of the response variables with localities from which the species were collected.

The second experiment on intra- and interspecific competition was evaluated by linear models (LMs), using deletion tests to obtain MAMs in the same way as described above. Stem height, branch number and shoot biomass were the response variables, and the fixed factorial treatment effects were species identity, intra- or interspecific competitors, and shade and moisture levels. To compare the strength of intra- with interspecific competition, after checking for insignificance of the three-level (species identity × competitor × shade × soil moisture) interaction, the individual species were evaluated separately, using for each of the four species the intraspecific competition as a reference level.

Some of the interactions among the explanatory variables were always significant, but the highest level interaction in the first (species identity × shade × soil moisture) and in the second experiment (species identity × competitor × shade × soil moisture) was always insignificant. Consequently, the individual species could be evaluated separately for each level of a factor appearing in a significant interaction. Using subsets of the whole data set particular for the significant interactions, these models were again simplified as described above, starting by the beyond optimal model for LMMs and maximal model for LMs, until MAMs were achieved. These MAMs were then visualised as bars of mean species values, separately for each level of the factor that appeared significant. For the models using shoot biomass as a covariate, in case of a parallel slope of the covariate on all species the MAMs were again visualised as bars of mean species values, expressed for the intercepts of the slopes of the covariate with x-axis. In case the slopes of the covariate for each species varied significantly, the MAMs were visualised as scatterplots of the response variable on the covariate, with a different slope for each species. In case the model could not be further simplified for the individual levels of the factors, we visualised just an overall pattern for these data, ignoring the treatments. Differences in mean values of the individual species were tested in LMs by least square differences (LSD) test, i.e. testing an *a priori* hypothesis that the mean values of the individual species significantly (P<0.05) differ (e.g. [69], p. 243). In LMMs the differences between species means were evaluated in deletion tests by collapsing the species factor levels (e.g. [67], p. 455–456).

Before analyses, branch number was square root+0.5, branch and peduncle biomass ln+0.5, and shoot, stem, and leaf biomass ln transformed to normalize the data (e.g. [69], p. 413–417)]. To adjust for multiply testing of the same data using a different response variable, we used a conservative Bonferroni correction (e.g. [69], p. 240). Consequently, for the repeated testing of the whole datasets with different response variables in the first experiment, only values of P<0.007, and in the second experiment on intra- and interspecific competition, only values of P<0.02 were considered significant at the 5% level of significance. Testing on subsets of the whole data in both experiments, including the LSD tests, was done at the conventional 5% level of significance, because each subset of data was used in analyses only once. All MAMs were checked for normality of residuals by making their histograms and normal probability (Q-Q) plots, and for homogeneity of error variance by plotting standardized residuals against explanatory variables, fitted values and individual levels of the explanatory variables (e.g. [65], p. 542–543). All calculations were done in R 2.9.2 [70], using the functions *lm* for linear models, and *lme* and *gls* for linear mixed effect models, with the latter function applied on LMMs with no random effect to enable including the models with no random effect in nested LR tests of random components ([65], p. 122).

## Results

### Experiment 1: Performance without competition

**Figure 1:**
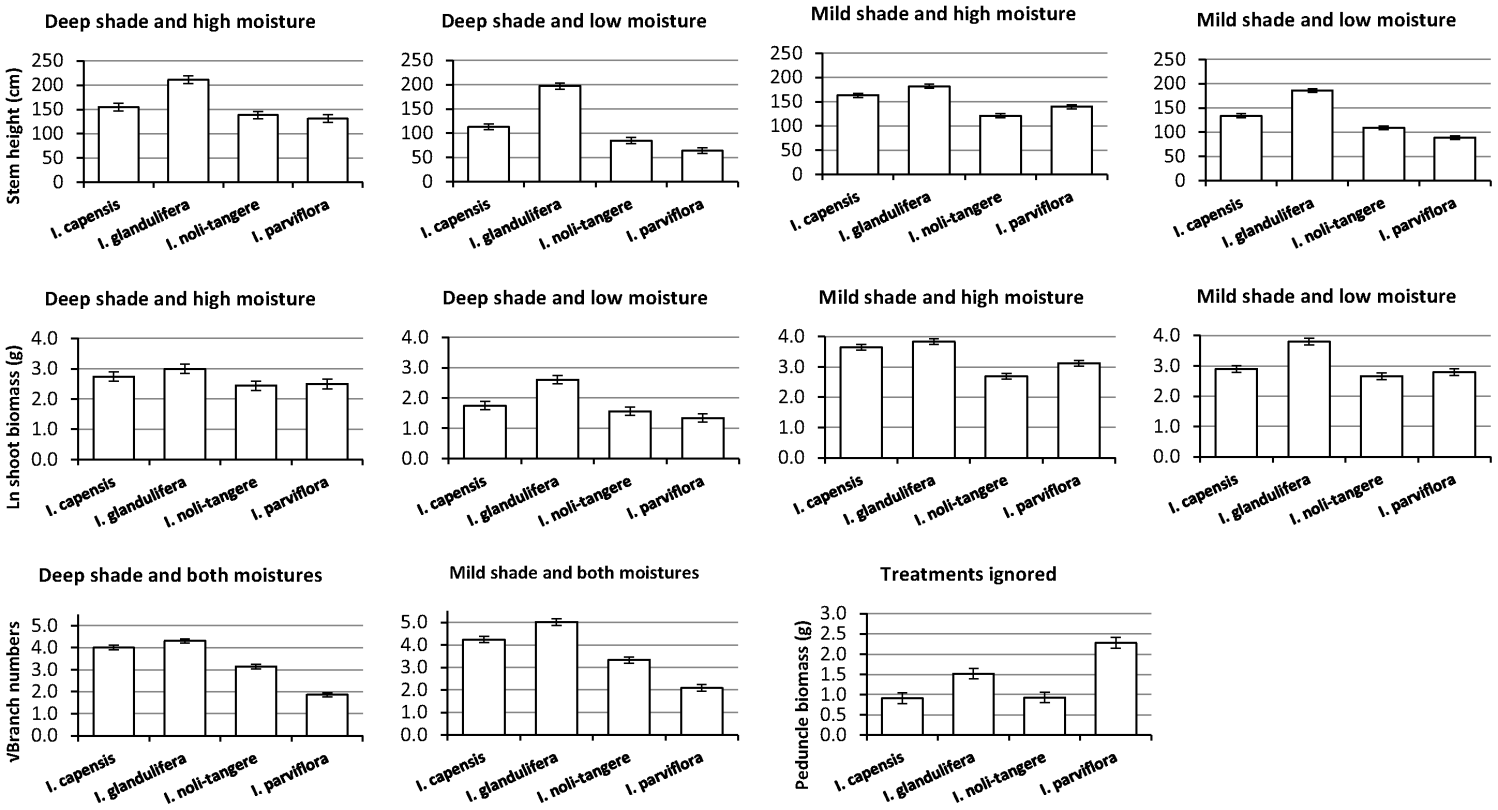
Comparison of stem height, shoot biomass, branch number, and peduncle biomass for *Impatiens capensis*, *I.* glandulifera, *I. noli-tangere* and *I. parviflora* in the first experiment. Note that because some values on peduncle biomass were negative on the logarithmic scale, they were back transformed to the original scale to ease visualisation; the LSD bars are slightly asymmetric due to the back transformation. Treatments are ignored if the highest level interaction for the analysed subset of the data was significant and the model thus could not be further simplified for the individual levels of the factors. Otherwise, the species are evaluated separately for each level of each factor that appeared significant. In case of significant interactions between the treatments, the pattern of bars differs for each level of each treatment. Bars of mean species values whose 95% least square differences (LSD) intervals do not overlap are significantly different.

When grown separately, *I. glandulifera* had the longest stems and the highest number of branches, followed by *I. capensis*. The ranking of *I. noli-tangere* and *I. parviflora* depended on the treatment, with non-significant differences in stem heights under deep shade and high moisture (Fig. 1). A similar pattern, although with less pronounced differences among the species, was observed in shoot biomass (Fig. 1). *Impatiens capensis* did not differ significantly from *I. noli-tangere* under deep shade and low moisture, from *I. glandulifera* under mild shade and high moisture, from *I. parviflora* under mild shade and low moisture, and from any of the congeners under deep shade and high moisture. There were no significant differences in shoot biomass between *I. parviflora* and *I. noli-tangere* except under mild shade and high moisture where *I. parviflora* was larger (Fig. 1). The highest investment into reproduction in terms of absolute or relative investment into peduncle biomass was found in *I. parviflora* (Fig. 1 and Figure S1). *Impatiens glandulifera* allocated the highest proportion of total biomass to stems (Figure S1) and the lowest to branches (Figure S1). *Impatiens noli-tangere* had the highest biomass of branches relative to other *Impatiens* species (Fig. S1). Biomass allocation into stems, branches and peduncles (the slopes of the covariate) varied significantly among the species in some treatments (Figures S1). Under mild shade and high moisture where all the species produced the greatest shoot biomass, stem biomass increased only slightly with increasing shoot biomass in *I. glandulifera*, but sharply in *I. capensis* (Figure S1).

Traits of all plant species showed remarkable plasticity depending on shade and soil moisture levels (Fig. 1). Stem height of *I. glandulifera* increased considerably under deep shade, while responding very slightly to soil moisture. *Impatiens noli-tangere* also tended to exhibit increased stem height in decreasing light conditions when coupled with abundant soil moisture, and decreased stem height in drier soil conditions. No marked response to shade levels was observed in *I. capensis* and *I. parviflora* under high soil moisture, while under low moisture deep shade decreased stem heights. Low levels of soil moisture resulted in decreased stem height in *I. noli-tangere*, *I. capensis* and *I. parviflora*. Branch numbers were significantly affected only by shade levels, with deep shade resulting in decreases for *I. glandulifera*, *I. capensis* and *I. parviflora*. Deep shade reduced shoot biomass of all species, its effect being the lowest on *I. noli-tangere* under high soil moisture. Low moisture decreased shoot biomass in all species under deep shade, but only of *I. capensis* and *I. parviflora* under mild shade.

**Figure 2:**
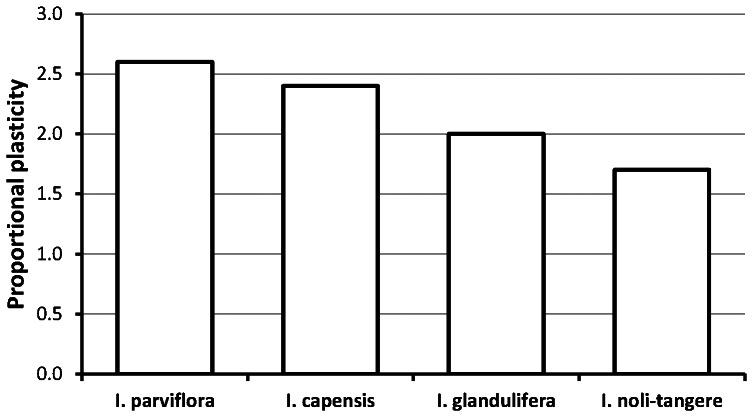
Species plasticity expressed as average proportional difference between the largest and the smallest value of stem height, shoot and relative leaf biomass in the first experiment for both levels of shade and soil moisture. Relative biomass was obtained from a model in which the total shoot biomass was fitted as a covariate.

As stem height, shoot biomass (Fig. 1) and proportional leaf biomass (Figure S1) were significantly influenced by both levels of shade and moisture, we calculated for these traits proportional plasticity, expressed as the proportional difference between the largest and the smallest average value of a given trait across all treatments, separately for each species (Fig. 2). The native species *I. noli-tangere* was the least plastic, and the most widespread invasive species *I. parviflora* was the most plastic (Fig. 2). *Impatiens parviflora* was the most plastic species in terms of stem height and shoot biomass (Fig. 1), and *I. capensis* in terms of relative leaf biomass (Figure S1). The potentially invasive species *I. capensis* was strongly limited by low moisture (Fig. 1).

Of the seven examined species traits, only the variance of three was significantly affected by the localities from which the seed used to grow plants in the experiment was collected: stem height (likelihood L = 7.645; df = 1, P<0.003), stem biomass (L = 35.577; df = 1; P<0.0001) and leaf biomass (L = 12.132; df = 1; P<0.001). Only the stem biomass had an intraclass correlation above 0.1 (ICC = 0.109; potential range of values 0–1), suggesting that the variation in this trait was associated with localities from which the species originated. This effect was most pronounced for the mild shade and low moisture treatment, producing the only minimal adequate model in which the effect of localities was also significant (L = 7.811, df = 1; P<0.003; ICC = 0.128). Intraclass correlations for stem high (ICC = 0.041) and leaf biomass (ICC = 0.044), although significant, were very low. No significant effect of the locality of origin was found for branch number, and shoot, peduncle and branch biomass.

### Experiment 2: Performance with competition

There is a clear pattern showing that the invasive species *I. glandulifera* and *I. parviflora* are the strongest competitors, the native species *I. noli-tangere* the weakest, and the potentially invasive species *I. capensis* is intermediate between the invasive and the native species. This pattern is most apparent with the synthetic measure of species performance, total shoot biomass (Table 1, Figure S2). The most invasive species, *I. glandulifera*, always significantly (P<0.05) reduced the shoot biomass of all congeners with which it was competing. The most widely distributed invasive species, *I. parviflora*, did not significantly reduce biomass of *I. capensis* and only marginally (0.05<P<0.1) reduced that of *I. glandulifera*, but it was the only species that performed better in intra- rather than inter-specific competition. Its shoot biomass was significantly higher if plants were grown together with those of *I. capensis* or *I. noli-tangere* than if grown with conspecific individuals (Table 1).

**Table 1:** Summary of comparisons of species traits (Stem height, Branch numbers and Shoot biomass) in the second experiment with four *Impatiens* species (*I. capensis*, *I. glandulifera*, *I. noli-tangere*, *I. parviflora*) under four environmental treatments (Deep/Mild shade and high/low moisture).

Trait	Species	Treatments	Competitors		
Stem height					
	I. capensis		glandulifera	noli-tangere	parviflora
		Ignored	0	0	0
	I. glandulifera		capensis	noli-tangere	parviflora
		Deep shade and high moisture	(↓)	0	0
		Deep shade and low moisture	0	0	↓
		Mild shade and high moisture	0	0	0
		Mild shade and low moisture	0	0	↓
	I. noli-tangere		capensis	glandulifera	parviflora
		Deep shade and both moisture levels	↓	↓	0
		Mild shade and both moisture levels	0	↓	↓
	I. parviflora		capensis	glandulifera	noli-tangere
		Ignored	0	↓	0
Branch numbers					
	I capensis		glandulifera	noli-tangere	parviflora
		Ignored	(↓)	0	↓
	I. glandulifera		capensis	noli-tangere	parviflora
		Deep shade and both moisture levels	0	0	0
		Mild shade and both moisture levels	0	0	0
	I. noli-tangere		capensis	glandulifera	parviflora
		Deep shade and high moisture	↓	↓	0
		Mild shade and high moisture	↓	↓	0
		Both shade levels and low moisture	0	↓	0
	I. parviflora		capensis	glandulifera	noli-tangere
		Deep shade and both moisture levels	0	0	0
		Mild shade and both moisture levels	0	0	0
Shoot biomass					
	capensis		glandulifera	noli-tangere	parviflora
		Ignored	↓	0	0
	glandulifera		capensis	noli-tangere	parviflora
		Deep shade and high moisture	0	0	(↓)
		Deep shade and low moisture	0	0	(↓)
		Mild shade and high moisture	0	0	(↓)
		Mild shade and low moisture	0	0	(↓)
	noli-tangere		capensis	glandulifera	parviflora
		Deep shade and high moisture	0	↓	↓
		Deep shade and low moisture	0	↓	↓
		Mild shade and high moisture	0	↓	↓
		Mild shade and low moisture	0	↓	↓
	parviflora		capensis	glandulifera	noli-tangere
		Deep shade and high moisture	↑	↓	↑
		Deep shade and low moisture	↑	↓	↑
		Mild shade and high moisture	↑	↓	↑
		Mild shade and low moisture	↑	↓	↑

The effect of intra- and interspecific competition is shown with the species used as the intraspecific competitor placed in the column Species and the interspecific competitors placed in the columns Competitors: ↓ means that the interspecific competitor has a significant (P<0.05) negative effect and ↑ that the competitor has a significant positive effect compared to intraspecific competition; arrows in brackets mark marginally (0.05<P<0.1) significant effects. Treatments are ignored if the highest level interaction for the analysed subset of the data was significant and the model thus could not be further simplified for the individual levels of the factors. Otherwise, the species are evaluated separately for each level of each factor that appeared significant. See Figures S2, S3, S4 for bars of mean species values with their 95% least square differences (LSD) intervals showing which species differences are significant.

The potentially invasive species *I. capensis* and the native *I. noli-tangere* never significantly reduced the biomass of their congeners. However, as a competitor, *I. capensis* significantly reduced the number of branches of the native *I. noli-tangere* under high moisture, and its stem height under deep shade and both moisture levels (Figure S3); under deep shade and high moisture it also marginally significantly reduced the stem height of *I. glandulifera* (Figure S4). On the contrary, as a competitor, the native *I. noli-tangere* never reduced any trait of the other congeners (Table 1, Figures S2–4).

## Discussion

### Impatiens glandulifera

Our greenhouse experiment revealed that plants of the highly invasive species *I. glandulifera* were taller than their congeners, regardless of the experimental treatment imposed. Together with the ability of its seedlings to grow tall under simulated canopy shade [38], the tall stature provides this species with a considerable advantage in terms of light acquisition. *Impatiens glandulifera* is thus a strong competitor against its shorter congeners, especially the native *I. noli-tangere*, which was suppressed by *I. glandulifera* in terms of all traits measured. *Impatiens parviflora* produced shorter stems and less shoot biomass in the presence of *I. glandulifera*. *Impatiens glandulifera* also reduced the shoot biomass of the second-tallest species, *I. capensis*. The tall stems of *I. glandulifera* are the likely reason for the absence of an interaction between shading and competitor identity in affecting shoot biomass of this species. However, it needs to be noted that while the species produced high biomass and tall stems under experimentally simulated deep shade, its performance under natural conditions may be reduced due to stem fragility (H. Skálová, personal observation). Possible physiological mechanisms underlying the competitiveness of *I. glandulifera* have not been described; however, it cannot be excluded that the reduction in photosynthetically active radiation, as well as radiation quality, could be involved, as reported for other invasive species [71]. The competitive advantage of *I. glandulifera* may be further supported by early germination in some years [63], which facilitates the efficient colonization of space during population development.

In adult plants as well as in seedlings [38] we observed a strong response to shade and a weak response to soil moisture. This weak response is probably the mechanism behind the generally low plasticity of this species revealed here. Strong response to shade provides an experimental explanation for the fact that the occurrence of *I. glandulifera* in the field is driven mostly by canopy cover. Its high competiveness and affinity for less-shaded sites seems to result in a shift of the realized niche of *I. noli-tangere* and *I. parviflora* into more-shaded areas when the species co-occur in the filed (J. Čuda et al., in prep.).

Both seedlings and adults of *I. glandulifera* performed well under low water availability, hence it is rather surprising that the species has been reported to require high soil moisture [55], [72]. However, this characteristic was suggested based on habitat preferences at a large scale rather than on ecological requirements in the field. Sites typically invaded by *I. glandulifera* are riparian habitats along streams that generally harbour high numbers of alien species [3], [73]–[75]. This is because such habitats provide suitable conditions for germination, establishment and growth of nutrient-demanding, fast-growing alien species [10], [76] and because water streams serve as vectors for propagules [73], [77], [78]. Our results suggest that in the case of *I. glandulifera*, the role water streams play in the dispersal of propagules may be more important than the affinity of the species to moist riparian habitats. Seeds of *I. glandulifera* are easily transported by water [79] and massive spread along rivers typically occurs shortly after the invasion, followed by later spread into more distant sites [52]. Considerable drought tolerance together with high demand for light can explain why this species has been recently colonizing habitats distant from river streams, such as woodland clearings and abandoned grasslands.

### Impatiens parviflora

The rather low biomass produced by *I. parviflora* indicates that the success of this species as an invader in the field is not related to the stature of mature plants. This suggests that the mechanism behind its successful invasion is linked with the seedling stage; seedlings of this species are larger than those of the other congeners [38]. The importance of the seedling stage for the invasion of this species is further emphasized by marked local differentiation reflected not only by seedling size and their growth response to environmental factors, but also by the timing of germination and frost resistance [49]. Besides seedling traits, investment in reproduction may also be associated with invasiveness of *I. parviflora*. Allocation to peduncle biomass, which was used as a proxy for reproductive effort in our study, was the highest of all the congeners tested. Interestingly, the very poor or even nonexistent seed bank (L. Moravcová and H. Skálová, personal observation) does not seem to limit its invasion. *Impatiens parviflora* seems to be a strong competitor under low soil moisture, causing a decrease in biomass of *I. glandulifera*. In addition, *I. parviflora* is the only species that performed better in inter- rather than intraspecific competition, and produced greater shoots if competing with *I. noli-tangere* and *I. capensis*. These results suggest that *I. parviflora* is a strong competitor relative to its congeners, especially to the native *I. noli-tangere*. Despite *I. parviflora* and *I. noli-tangere* producing comparable biomass if grown separately, the biomass of the former was considerably higher than that of the latter if grown together.

### Impatiens capensis

As in previous studies [38], [49], [63] the performance of *I. capensis* was within the range of the other *Impatiens* species studied. This indicates that further spread of *I. capensis* into central and eastern Europe may be possible. However, the present study indicates that there may be also some biological and ecological constraints. The growth of this species appears to be limited by the presence of *I. glandulifera*, which considerably decreased shoot biomass of *I. capensis* compared to intraspecific competition, and by that of *I. parviflora*, which reduced the branching of *I. capensis*. In addition, the species has small seedlings that may also reduce its success at the initial stage of population development [38]. Thus the potential to invade seems less likely than that of *I. glandulifera*. Overall, the other congeners were suppressed by *I. capensis* to a lesser degree than by the two species that are currently invasive in the study region. This indicates that large size of an alien plant species may not necessarily result in its invasion success, and ability to compete within the range of environmental conditions may be a more important factor.

### Comparison of invasive and native species

As with the previous study addressing seedling traits [38], the present results indicate that the invasive species grow larger and exhibit higher plasticity in size traits than the native *I. noli-tangere*. *Impatiens noli-tangere* also appears to be a weaker competitor than all the three invasive species tested, and did not suppress any of them in competition. This indicates that local competitive exclusion of *I. noli-tangere* by its alien congeners seems likely because the niches of the native and invasive *Impatiens* species overlap to a great degree. The poor performance of *I. noli-tangere* under interspecific competition is another example of a native species being affected by competition with alien species [80]. On the other hand, *I. noli-tangere* seems to be more resistant to damaging events than its congeners. It is more resistant to frosts [49] and unlike the congeners it forms a seed bank that may ensure population recovery after damaging events [63]. This suggests that poor competitive ability of the native species, which may lead to population declines under inter-specific competition with its invasive congeners, may be to some degree overcome by its higher tolerance of natural constraining factors.

## Supporting Information

Figure S1 **Comparison of stem biomass, branch biomass, leaf biomass and peduncle biomass for** ***Impatiens capensis*** **(C),** ***I. glandulifera*** **(G),** ***I. noli-tangere*** **(NT) and** ***I. parviflora*** **(P) in the first experiment with the total shoot biomass as a covariate.** Treatments are ignored if the highest level interaction for the analysed subset of the data was significant and the model thus could not be further simplified for the individual levels of the factors. Otherwise, the species are evaluated separately for each level of each factor that appeared significant. In case of significant interactions between the treatments, the pattern of bars differs for each level of each treatment. Bars of mean species values whose 95% least square differences (LSD) intervals do not overlap are significantly different. In case of a parallel slope of the covariate on all species the patterns are visualised with bars of mean species values expressed for the intercepts of the slopes of the covariate with x-axis; these intercepts correspond to log one of particular biomasses. In case the slopes of the covariate for each species varied significantly, the patterns are visualised as scatterplots of the response variable on the covariate, with a different slope for each species. The panel in (A) without LSD intervals is calculated from LMM model; bars with different letters are significantly (P<0.05) different.
(EPS)

Figure S2 **Comparisons of shoot biomass of I. capensis, I. glandulifera, I. noli-tangere and I. parviflora in the second experiment.** Otherwise as in Fig. S1.
(EPS)

Figure S3 **Comparisons of branch number of I. capensis, I. glandulifera, I. noli-tangere and I. parviflora in the second experiment.** In case that the treatments are significant but do not interact, the height of bars is different for each level of the significant treatment but the pattern of bars is the same for all species (B). In (C), both the effect of shade and soil moisture are significant, but only the interaction of the species with soil moisture is significant. Otherwise as in Fig. S1.
(EPS)

Figure S4 **Comparisons of stem height of I. capensis (A), I. glandulifera (B), I. noli-tangere (C) and I. parviflora (D) in the second experiment.** The effect of intra- and interspecific competition on stem height is visualised with the species used as the intraspecific competitor placed first on x-axis (A: C, Impatiens capensis; B: G, I. glandulifera; C: NT, I. noli-tangere; D: P, I. parviflora), and the interspecific competitors as the further three species on x-axis. Treatments are ignored if the highest level interaction for the analysed subset of the data was significant and the model thus could not be further simplified for the individual levels of the factors. Otherwise, the species are evaluated separately for each level of each factor that appeared significant. In case of significant interactions between the treatments, the pattern of bars differs for each level of each treatment. Bars of mean species values whose 95% least square differences (LSD) intervals do not overlap are significantly different.
(EPS)
